# EEG resolutions in detecting and decoding finger movements from spectral analysis

**DOI:** 10.3389/fnins.2015.00308

**Published:** 2015-09-01

**Authors:** Ran Xiao, Lei Ding

**Affiliations:** ^1^School of Electrical and Computer Engineering, University of OklahomaNorman, OK, USA; ^2^Biomedical Engineering Center, University of OklahomaNorman, OK, USA

**Keywords:** spectral features, fine body-part movement, EEG, PCA, BCI

## Abstract

Mu/beta rhythms are well-studied brain activities that originate from sensorimotor cortices. These rhythms reveal spectral changes in alpha and beta bands induced by movements of different body parts, e.g., hands and limbs, in electroencephalography (EEG) signals. However, less can be revealed in them about movements of different fine body parts that activate adjacent brain regions, such as individual fingers from one hand. Several studies have reported spatial and temporal couplings of rhythmic activities at different frequency bands, suggesting the existence of well-defined spectral structures across multiple frequency bands. In the present study, spectral principal component analysis (PCA) was applied on EEG data, obtained from a finger movement task, to identify cross-frequency spectral structures. Features from identified spectral structures were examined in their spatial patterns, cross-condition pattern changes, detection capability of finger movements from resting, and decoding performance of individual finger movements in comparison to classic mu/beta rhythms. These new features reveal some similar, but more different spatial and spectral patterns as compared with classic mu/beta rhythms. Decoding results further indicate that these new features (91%) can detect finger movements much better than classic mu/beta rhythms (75.6%). More importantly, these new features reveal discriminative information about movements of different fingers (fine body-part movements), which is not available in classic mu/beta rhythms. The capability in decoding fingers (and hand gestures in the future) from EEG will contribute significantly to the development of non-invasive BCI and neuroprosthesis with intuitive and flexible controls.

## Introduction

Rhythmic brain activities, biomarkers of many important brain functions, have been long studied with magnetic and electrical signals, i.e., magnetoencephalography (MEG) and electroencephalography (EEG). These activities are believed due to aggregated neural oscillations, which suggest various brain states under either resting or tasked conditions (Steriade et al., 1990). The most well-known rhythmic activity arising from the human brain is the alpha wave (i.e., 8–12 Hz), which can be observed at the occipital area during wakeful relaxation with eyes closed and is reduced with eyes open (Berger, 1933; Kirschfeld, 2005). The alpha wave is considered as an idle state of the visual function and its variations serve as an indicator of functional change in the visual cortex related to, such as sleep (McKinney et al., 2011) and drowsiness (Lin et al., 2005). Another important rhythmic activity is the theta rhythm (i.e., 4–7 Hz) that is associated with memory processing when it appears in the frontal cortex (Urgen et al., 2013) and spatial navigation when in the parietal cortex (Snider et al., 2013). Recently, rhythmic high-frequency oscillations (HFO, i.e., 40 Hz and higher) have gained increasing attention (Gotman, 2010; Jacobs et al., 2012; Worrell, 2012). In the clinical field, the emergence of some pathophysiological HFOs has been spatially and temporally accompanied with seizure onsets in neocortical epileptic patients (Worrell et al., 2004; Jirsch et al., 2006; Jacobs and Kahana, 2009). Therefore, rhythmic activities observed in EEG and MEG not only serve as a gateway to understand underlying neuronal mechanisms, but also provide valuable pathological information that can be used to address clinical problems.

In the human motor cortex, one of well-studied brain oscillations is the mu rhythm at the alpha band (i.e., 8–12 Hz) (Pfurtscheller and Lopes da Silva, 1999; Pfurtscheller et al., 2006; Yuan and He, 2014). The attenuation of the alpha band power can be observed during preparation and/or execution of voluntary movements, which is accompanied by the beta band (i.e., 13–30 Hz) power decrease, known as event-related desynchronization (ERD) (Pfurtscheller and Lopes da Silva, 1999). Furthermore, ERD is usually followed by a power rebound in the beta band after cessation of movements, known as event-related synchronization (ERS). These phenomena reflect the change of synchrony in underlying neuron populations in the motor brain (Pfurtscheller and Lopes da Silva, 1999). It has also been demonstrated that healthy people and disabled patients, through training, can deliberately control the mu rhythm power by imagining different types of movements (Pfurtscheller et al., 2006; Silvoni et al., 2011), which have been utilized to control man-made brain-computer interfaces (BCI) in driving, e.g., wheel chair (Huang et al., 2012) or computer programs (Wolpaw and McFarland, 2004; Wilson et al., 2009). In the motor cortex, high-frequency gamma oscillations (over 70 Hz) have also been observed in both non-invasive (Darvas et al., 2010) and invasive EEGs, i.e., electrocorticography (ECoG, Crone et al., 1998; Miller et al., 2007). These studies find an elevation of the gamma power over the primary motor cortex during movements of finger or other body parts. Aside from the difference in frequency ranges, low- (i.e., mu/beta) and high-frequency rhythms (i.e., gamma) also distinguish each other regarding their spatial and temporal patterns. Low-frequency rhythms are typically observed bilaterally during unilateral movements and show decreased power throughout movement periods, while movement-related high-frequency rhythmic increases are highly time-locked to movement onset and observed only in the contralateral motor cortex during unilateral movements (Cheyne et al., 2008). Furthermore, high-frequency rhythms indicate more detailed somatotopic organization spatially over the sensorimotor areas than low-frequency mu/beta rhythms. Features from high-frequency bands have been successfully used to decode movements of fine body parts, e.g., fingers (Miller et al., 2009; Liao et al., 2014) and wrist (Khan and Sepulveda, 2010), while low-frequency components are mostly applied to decode movements of large body parts, e.g., hand and foot (Neuper and Pfurtscheller, 1996; Wolpaw and McFarland, 2004; Hashimoto and Ushiba, 2013).

While aforementioned rhythmic activities represent frequency-specific changes in ongoing brain signals, recent studies further reveal that rhythmic activities at different frequency bands suggest spatial and temporal couplings (Pfurtscheller et al., 1997; Canolty et al., 2006; Miller et al., 2009). In the motor brain, the non-linear couplings among harmonic frequency components between mu and beta rhythms and between low and high beta rhythms have been reported (Pfurtscheller et al., 1997; Pfurtscheller and Lopes da Silva, 1999). Coupling between high gamma power and theta oscillation has also been observed in cognitive processes of the human brain studied using ECoG signals (Canolty et al., 2006). One clinical study further indicates the coexistence of slow shift and high frequency oscillation during seizures in epileptic patients (Imamura et al., 2011). A recent study (Miller et al., 2009) reported power increase over a broadband spectrum (up to 200 Hz) in a finger tapping task, obtained by principal component analysis (PCA) on ECoG spectral data. These studies demonstrate that well-defined spectral structures over multiple frequency bands might exist in brain signals and their changes may contain rich information that is not available in the analysis of rhythmic activities at individual frequency bands. However, most of phenomena in the spectral domain of brain signals are still obtained through the Fourier analysis and interpreted at individual frequency bins or bands. Less has been conducted to explore spectral structures of brain signals than their spatial and temporal structures.

The aim of the present study is to directly investigate spectral structures in non-invasive EEG data through the use of PCA on spectral covariance matrix of data from motor tasks performing individual finger movements. Our hypothesis is the broadband and other spectral structures observed in ECoG data (Miller et al., 2009, 2014) related to finger movements can be recovered in non-invasive EEG data as well, and we further expect that the identification of these spectral structures might provide more valuable information about the motor brain, e.g., better characterization of somatotopic organization within the sensorimotor cortex. In contrast to rhythmic activities obtained through classic analysis, i.e., mu/beta rhythms, we demonstrated the similarity and difference between these newly identified spectral structures and classic rhythmic activities. Our results indicated significantly improved performance in detecting movements of individual fingers from resting using new spectral structure features as compared with classic rhythmic features. We further investigated the characteristics of these spectral structure features and studied their efficacies in decoding individual finger movements from one hand. Different combinations of both types of features were also evaluated, aiming to investigate information independence and redundancy in different categories of features. Our experimental results indicated promising potentials of the newly identified spectral structures from EEG data in decoding movements of fine body parts, which could facilitate the development of non-invasive BCI and neuroprosthesis (Pfurtscheller et al., 1995; Guger et al., 2000; Müller-Putz et al., 2005). Some of preliminary results focusing on decoding finger movements in fewer subjects rather than comprehensive investigation of these new features have been reported in Xiao and Ding (2013).

## Materials and methods

### Experimental protocol

EEG data were recorded from 11 subjects (mean age: 26.4 years, range: 22–32 years, all right handed), with written informed consent from all subjects. The study was approved by the Institutional Review Board of the University of Oklahoma. Data from one subject were excluded due to poor recording quality. A 128-channel EEG system (Net Amps 300, Electrical Geodesic Inc., OR, USA) was used to acquire EEG signals at sampling rate of either 250 or 1000 Hz (down sampled to 250 Hz later) and all channels were referenced to a non-data channel at vertex.

The experiments were conducted in a dimly lighted and shielded chamber room. Subjects sit in a comfortable armchair with their arms supported and relaxed. A LCD screen was placed in front of them to display visual cues, which were designed using the E-Prime software (Psychology Software Tools, Inc. Pittsburgh, PA, USA). Each trial lasted for 6 s. In the first 2 s, the screen was blank. In the following 2 s, a fixation appeared in the middle of the screen to indicate upcoming movement cues. Subjects were instructed to gaze at the fixation without movements to prepare for upcoming tasks. After that, one of five wording cues (i.e., thumb, index, middle, ring, and little) was randomly presented for 2 s. Subjects were asked to perform continuous flexion and extension of the corresponding finger on the right hand, while most of them finished two rounds of flexion and extension in 2 s. There were eighty 6-s trials for each finger, i.e., 400 trials in total for five fingers in the entire session, finished by most subjects (300 trials by one subject). During experiments, actual movements were monitored through a camera in real time in order to identify trials with wrong finger moved, and these trials were removed later from analysis.

### Preprocessing

Datasets recorded at the sampling rate of 1000 Hz were firstly downsampled to 250 Hz to be consistent with other datasets. The first 2-s EEG data in each 6-s trial were removed from further analysis, since the period was designed for subjects to engage unavoidable movements, such as blink or swallowing. The remaining data were then high-pass filtered at 0.3 Hz using an elliptic infinite impulse response (IIR) filter from the EEGLAB toolbox (Delorme and Makeig, 2004) with both forward and reverse filtering to minimize phase distortions. A 60 Hz notch filter with a transition band of 0.3 Hz was further applied to remove power-line noise. To remove common physiological artifacts, independent component analysis (ICA) (Hyvärinen et al., 2001) from the EEGLAB toolbox was performed, implemented with the Infomax algorithm (Bell and Sejnowski, 1995). EEG artifacts, such as generic discontinuities, electrooculogram (EOG), electrocardiogram (ECG), and electromyogram (EMG), were then identified and rejected using the ADJUST toolbox (Mognon et al., 2011) and visual inspections. Total 64 independent components (ICs) were reconstructed and about 10–20 artifact-related ICs were rejected in each subject.

To further increase signal-to-noise ratio (SNR), EEG signals went through a common average reference (CAR) filter (McFarland et al., 1997), with data from each channel re-referenced to the average of data from all channels. After these steps, the 1-s segments of EEG data in the middle of 2-s period for fixation and 2-s period for movement in each trial were selected for the following analysis. This resulted in total six conditions: EEG data of five finger-movement conditions and pooled EEG data from resting conditions (i.e., fixation periods). It led to 60 ~ 80 one-second movement segments and about five times of resting segments in each subject.

### Spectral analysis

Spectral patterns of brain signals related to the motor tasks were examined through spectral analysis. EEG temporal data were firstly transformed into the frequency domain by calculating power spectral density (PSD) at each channel and all segments from both movement and resting conditions, using

(1) $$\begin{array}{l} P_{n}^{m}(f)=\frac{1}{T}{\left|\sum_{t\;=\;1}^{T}X_{n}^{m}\left(t\right)\cdot H(t)\cdot \exp\left(i\frac{2\pi }{T}(f-1)t\right)\right|}^{2},\\ \;f=1,2,\cdots ,70\text{Hz},\;m=1,2,\cdots ,M \end{array}$$

where *X^m^_n_*(*t*) is the temporal EEG data in the 1-s segment *m* on channel *n*, and *P^m^_n_*(*f*) is the corresponding PSD at frequency *f*. *M* is the total number of segments including all finger movement and resting segments, and *T* is the sampling frequency. *H*(*t*) is the Hanning window, i.e., *H*(*t*) = (1 + cos(2π*t*/*T*))/2, used to minimize power leakages in spectral power calculation. In the analysis of classic motor rhythms, the magnitudes of PSDs in alpha and beta bands were obtained to examine spectral changes between movements and resting and between different types of movements (Babiloni et al., 1999; Pfurtscheller et al., 2006). To probe new spectral structures in EEG, the spectral PCA analysis detailed below was applied (Miller et al., 2009).

Firstly, log normalization was performed on each segment to scale power increase (between zero to infinity after logarithm) and power decrease (between negative infinity to zero) equally with respect to the mean of all segments (Miller et al., 2009), by:

(2) $$\begin{array}{l} \tilde{P_{n}^{m}}(f)=\ln\left(P_{n}^{m}(f)\right)-\ln\left(\frac{1}{M}\sum_{m\;=\;1}^{M}P_{n}^{m}(f)\right),\\ \;f=1,2,\cdots 70\text{Hz} \end{array}$$

where $\tilde{P_{n}^{m}}(f)$ is the log normalized spectral powers at frequency *f*on channel *n* and segment *m*.

Secondly, a channel-wise PCA analysis was performed on the log-normalized spectral data to identify common spectral structures across all conditions (Glaser and Ruchkin, 1976). The covariance matrix of PSD data over the whole-band frequency range was constructed by:

(3) $$\begin{array}{l} C(f,\;f^{\prime })=\sum_{m}\tilde{P_{n}^{m}}(f)\cdot \tilde{P_{n}^{m}}(f^{\prime }),\;f,\;f^{\prime }=1,2,\cdots ,70\text{Hz},\\ \;m=1,2,\cdots ,M \end{array}$$

where *C*(*f, f*′) is the covariance matrix of PSDs as a function of frequency. Its eigenvalues and eigenvectors were calculated and denoted by λ_*k*_ and *e*_*k*_, where *k* = 1, 2, ⋯, 70. The sequence of eigenvectors *e*_*k*_ (principal components, or PCs) was arranged by the values of their corresponding eigenvalues λ_*k*_ in a descending order. These spectral PCs represented different common spectral structures in EEG across conditions, ordered with decreasing significance.

Lastly, the PSD data from each segment were projected onto different spectral PCs:

(4) $$W_{n,k}^{m}=\sum_{f}e_{k}(f)\cdot {\tilde{P}}_{n}^{m}(f),\;f=1,2,\cdots ,70\text{Hz}$$

where *W^m^_n,k_* is the projection weight of segment *m* at channel *n* on *k*^*th*^ PC. These projection weights were used as spectral features to evaluate changes in spectral structures under different conditions as discussed below.

### Evaluation of spectral features

The evaluation of new spectral features consisted of two parts: qualitative inspection of their characteristic spectral profiles and spatial patterns, and quantitative assessment of their efficacies in distinguishing finger movements against resting and movements of different fingers using confusion matrices based on decoding accuracy data. These evaluations were performed in comparison to classic mu/beta rhythms, and detailed below.

Firstly, topographies of spectral features associated with the first three spectral structures (accounted for most data variance) were compared with topographies of mu/beta rhythms via visual inspections in conditions of resting and movements of different fingers. The topographies of mu/beta rhythms were obtained by mapping averaged spectral powers within each frequency band on the scalp.

Secondly, cross condition changes (i.e., resting vs. movement and movements of different fingers) in spatial patterns of mu, beta, and three new spectral features were quantitatively evaluated using coefficient of determination (*r*^2^ values):

(5) $$r=\frac{\sqrt{n_{1}\cdot n_{2}}}{n_{1}+n_{2}}\cdot \frac{mean(w_{1})-mean(w_{2})}{std(w_{1}\cup w_{2})},\;r^{2}=r\cdot r$$

where *n*_1_ and *n*_2_ are numbers of segments for two conditions to be compared. *w*_1_ and *w*_2_ are the feature vectors of each condition, which are data defining spatial patterns of features. They are projection weights on spectral PCs at channels for the three new spectral features, and PSDs in the alpha and beta bands at channels for the mu/beta spectral features. The *std*(*w*_1_⋃*w*_2_) calculates the standard deviation of data pooled together from two conditions. The calculation of *r*^2^ values was performed between two conditions of same features at channels and, therefore, topographies of differences for different features and conditions were generated.

Lastly, two types of spectral features (i.e., projection weights on the first three PCs and alpha/beta band powers) were evaluated in two decoding tasks involving individual finger movements. In the first task, movements of five fingers were grouped as the movement condition to be decoded from the resting condition. The second task was to decode movements of five fingers to create the confusion matrix of five fingers for each feature. Both decoding tasks were also performed using spectral features of mu/beta rhythms. Furthermore, to study the independence and redundancy of information in different features in detecting movements and decoding different finger movements, various combinations of spectral features (e.g., three PCs; mu+beta; and mu+beta+three PCs) were also investigated for both tasks. One-sample Student's *t*-test was performed to evaluate whether decoding accuracy is significantly higher than the guessing level in each decoding task. And paired Student's *t*-test was performed to compare decoding accuracies from using different spectral features.

### Classification procedures

Classification procedures for evaluation of spectral features through the two decoding tasks discussed above are described here. Since most EEG features exhibited localized spatial patterns (e.g., mu/beta rhythms over the motor cortex), spectral features from subsets of all channels were used as input features to classifiers to avoid negative impacts from irrelevant channels. For the classic mu/beta rhythms features, channel C3 and its neighboring channels were chosen as feature channels (for right hand movements). For features from the PCs, channels were selected based on *r*^2^ values between two compared conditions. In general, channels were ranked by their corresponding *r*^2^ values, and then the first 10 channels were chosen as feature channels. If more than two conditions to be compared, i.e., five fingers, the union of selected channels for all finger pairs was used. For cases using combined features, the union of selected channels for each feature was used.

The linear support vector machine (SVM) (Vapnik, 1998, 1999) with radial basis function (RBF), implemented in a MATLAB package, i.e., LIBSVM (Chang and Lin, 2011), was chosen for classification. The penalty parameter and gamma value in the RBF kernel were determined by a grid-search approach within the range of logarithm value [−10, 20] and [−15, 10], respectively, with the step width of one (Hsu et al., 2003). The decoding features (projection weights on PCs, and mu/beta PSDs) were linearly scaled into the range [−1, +1] to avoid numeric range dominance of one feature over others. A binary SVM classifier was applied in detecting movements from resting and the one-vs.-one scheme followed by a majority voting was used to solve the multiclass classification problem (Hsu and Lin, 2002) in decoding five fingers. A five-fold cross validation procedure was implemented, i.e., 80% data for training and 20% data for testing, which was repeated 30 times by randomly partitioning data into training and testing sets.

## Results

### Spectral structures from the spectral PCA analysis

**Figure 1:**
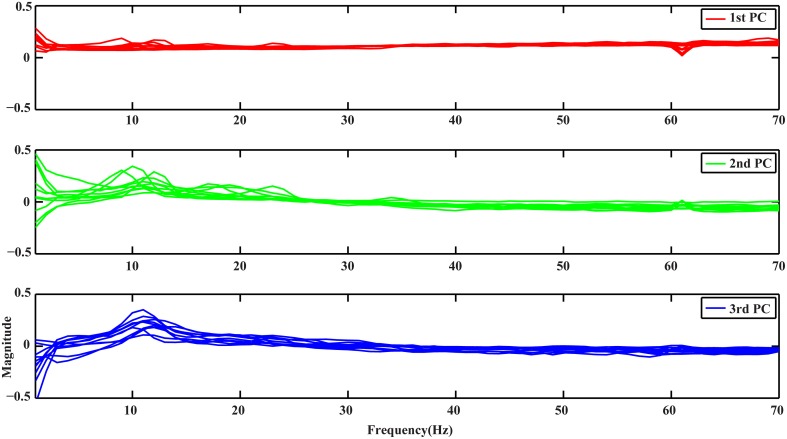
**Spectral profiles of the 3 PCs from all subjects obtained by PCA**. Red, green, and blue curves represent profiles of the 1st, 2nd, and 3rd PCs, respectively.

Figure 1 depicts the profiles of first three PCs from the spectral PCA analysis, with each curve representing spectral structure derived from one subject in all plots. It is noted that all curves in each PC present similar patterns, suggesting the consistency of these spectral structures across subjects, while different PCs show distinct profiles along the whole frequency range (i.e., 1–70 Hz). The 1st PC (red curves) is generally flat with positive elevations across the whole frequency range, which reveals a broadband phenomenon. The 2nd PC (green curves) presents spectral peaks at both alpha and beta bands while exhibits values close to zero for other frequency bands. The 3rd PC (blue curves) presents main peaks at the alpha band.

### Spatial patterns of new spectral features

**Figure 2:**
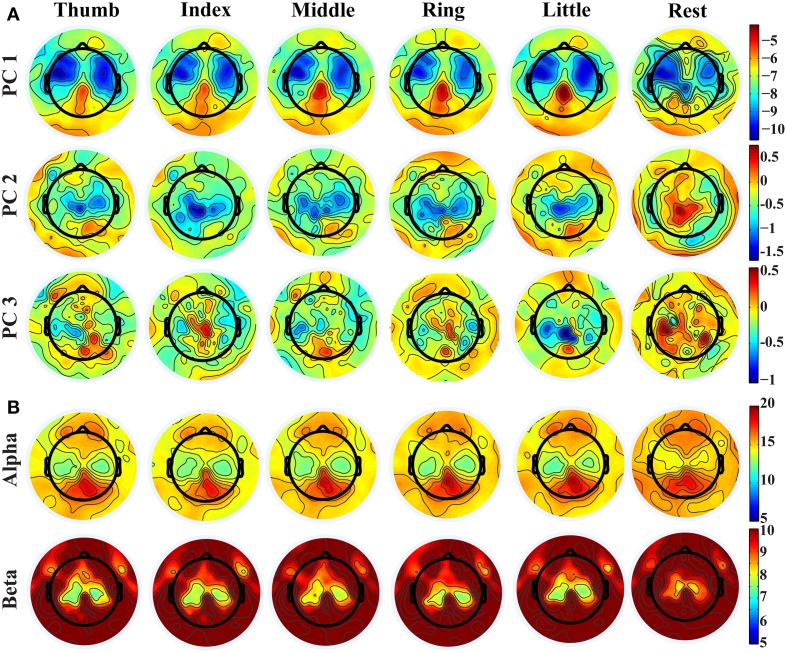
**(A)** Topographies of projection weights on different PCs for conditions of different finger movements and resting. **(B)** Topographies of PSDs in alpha and beta bands.

**Figure 3:**
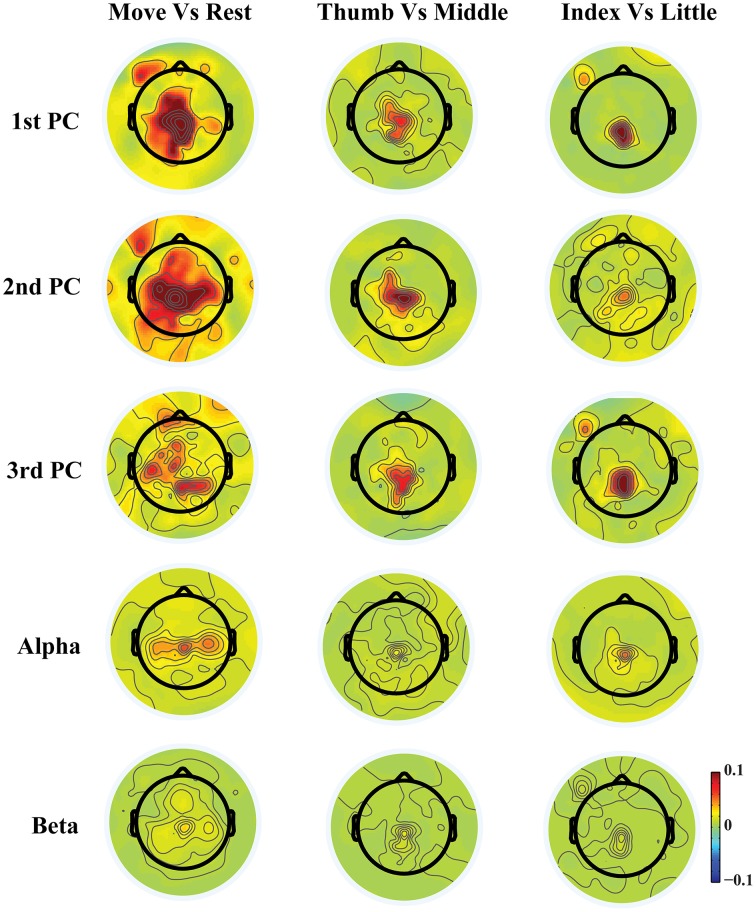
**Topographies of *r*^2^ values between movements and resting, between movements of thumb and middle, and between movements of index and little**.

Distinct spatial patterns are observed in the distributions of projection weights on PCs, as shown in Figure 2A. The first row shows the averaged topographies of projection weights over all subjects on the 1st PC from different fingers. Bilateral clusters of large projection weights (e.g., around −10) are observed over the primary motor (M1) and premotor cortices, which extend more toward anterior areas of the brain. And smaller projection weights (e.g., around −4) form an outstanding cluster in the posterior parietal area. These brain areas, especially the parietal area, also indicate differences when comparing projection weights from movements and resting. Major clusters of projection weights on the 2nd PC are mainly over the central area (the second row in Figure 2A), including M1 and supplementary motor area (SMA), which also show significant difference between finger movements and resting (with sign changes). Unlike the first two PCs, the 3rd PC indicates scattered patterns in distributions of projection weights, while some relatively weak patterns can still be observed over the central and parietal areas when movement conditions are compared with resting. Mu/beta powers (Figure 2B) show decreasing patterns during movements as compared with resting over bilateral M1, which is consistent with previous studies (Pfurtscheller, 1989; Magnani et al., 1998; Szurhaj et al., 2001) and similar to bilateral patterns over M1 in both the 1st and 2nd PCs. Mu/beta powers (especially mu power) also indicate a clustered pattern over the central parietal area, similar to what is observed in the 1st PC, while its changes between movements and resting are not as large as in the 1st PC (see Figure 3 also). It is noted that, while some similarities are observed between the PCs and mu/beta powers, many differences are also suggested when whole patterns of individual features are compared one to another.

Figure 3 shows exemplary scalp maps of *r*^2^ values, which provide quantitative metrics for cross-condition differences in individual features. Broader differences over M1, SMA, premotor, and parietal areas from three PCs are observed in the comparison between movements and resting, while more focused differences over SMA, parietal, and some left M1 areas are shown in the comparison of different finger movements. It is observed that some areas that indicate large projection weights (e.g., bilateral premotor and anterior areas in the 1st PC) show almost no changes across different fingers. It is also suggested that much more differences between different conditions are revealed from three new spectral features than mu/beta features. In particular, both mu and beta powers show almost no difference for movements of different fingers.

### Resolutions of spectral features in detecting movements from resting

**Figure 4:**
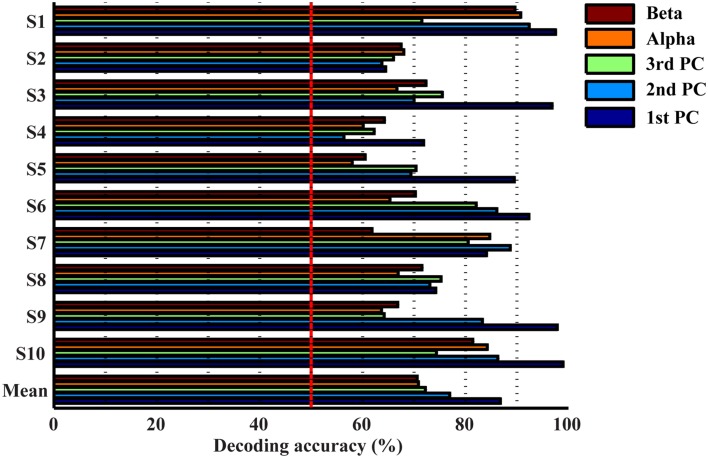
**Accuracies in detecting movements from resting using individual features**.

Figure 4 presents the accuracy in decoding movements from resting using mu, beta, and spectral features from PCs. It indicates that all features individually yield significantly higher detection accuracy than the guessing level (*p* < 0.05), suggesting the existence of spectral changes in EEG associated with movements. The mean decoding accuracy achieved by the spectral feature from the 1st PC is 86.8%, followed by the 2nd PC at 76.9% and the 3rd PC at 72.2%, indicating that all three PCs contain discriminative information of finger movements from resting. Spectral powers on the alpha (70.8%) and beta bands (70.6%) yield lower decoding accuracy than all individual PCs, and significantly lower than the 1st PC (*p* < 0.05, Table 1).

**Table 1:** **Summary of Student *t*-test results (*p*-values) among decoding accuracies of movements from resting condition using different features**.

	**1st PC**	**2nd PC**	**3rd PC**	**2 PCs**	**3 PCs**	**Alpha (A)**	**Beta (B)**	**A+B**	**A+3PCs**	**B+3PCs**	**AB+3PCs**
1st PC	NA	**0.0111**	**0.0043**	−**0.0193**	−**0.0084**	**0.0055**	**0.0017**	**0.0125**	−**0.0206**	−**0.0166**	−**0.0212**
2nd PC	−**0.0111**	NA	0.1643	−**0.0004**	−**0.0002**	0.0539	0.0954	0.5643	−**0.0001**	−**0.0001**	−**0.0001**
3rd PC	−**0.0043**	−0.1643	NA	−**0.0003**	−**0.0001**	0.7009	0.6402	−0.2042	−**0.0000**	−**0.0000**	−**0.0000**
2 PCs	**0.0193**	**0.0004**	**0.0003**	NA	−0.0619	**0.0011**	**0.0006**	**0.0017**	−0.2609	−0.2487	−0.2236
3 PCs	**0.0084**	**0.0002**	**0.0001**	**0.0619**	NA	**0.0006**	**0.0002**	**0.0006**	−0.8308	−0.5863	−0.5667
Alpha (A)	−**0.0055**	−0.0539	−0.7009	−**0.0011**	−**0.0006**	NA	0.9324	−**0.0036**	−**0.0003**	−**0.0002**	−**0.0002**
Beta (B)	−**0.0017**	−0.0954	−0.6402	−**0.0006**	−**0.0002**	−0.9324	NA	**0.0247**	−**0.0001**	−**0.0001**	−**0.0001**
A+B	−**0.0125**	−0.5643	0.2042	−**0.0017**	−**0.0006**	**0.0036**	**0.0247**	NA	−**0.0002**	−**0.0001**	−**0.0001**
A+3PCs	**0.0206**	**0.0001**	**0.0000**	0.2609	0.8308	**0.0003**	**0.0001**	**0.0002**	NA	−0.5669	−0.5020
B+3PCs	**0.0166**	**0.0001**	**0.0000**	0.2487	0.5863	**0.0002**	**0.0001**	**0.0001**	0.5669	NA	−0.9870
AB+3PCs	**0.0212**	**0.0001**	**0.0000**	0.2236	0.5667	**0.0002**	**0.0001**	**0.0001**	0.5020	0.9870	NA

*The bold and underlined entries indicate significant difference (p < 0.05). Negative entries indicate low decoding accuracy using the feature from row than the one from column*.

**Figure 5:**
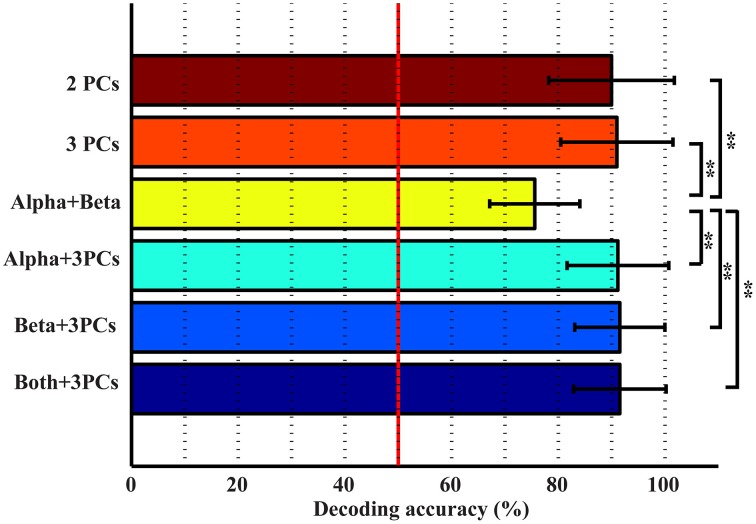
**Accuracies in detecting movements from resting using combined features**. ^**^p < 0.01.

The top three bars in Figure 5 present the decoding accuracy using combined features from only one category of spectral features (projection weights on spectral PCs or PSDs). It is observed that two or three spectral PCs together produce significantly higher decoding accuracy, i.e., 90 and 91%, respectively, than individual PCs (*p* < 0.05 for the 1st PC and *p* < 0.0005 for the 2nd and 3rd PCs, Table 1). Similar phenomenon is also observed for the combined alpha and beta bands feature, in which the decoding accuracy (i.e., 75.6%) is significantly higher than the feature only from either alpha or beta band alone (*p* < 0.05, Table 1). Moreover, the combined features from the spectral PCs as the input feature for classification show much higher accuracy than the combined PSD features (*p* < 0.001, Table 1). On the other hand, when features from different categories are combined (spectral PCs and PSDs), only slight improvements in decoding accuracy are observed (91.5% by combining total five features), which are not significantly different from ones obtained through the use of combined spectral PCs (i.e., 91% for combined three PCs).

### Resolutions of spectral features in decoding individual finger movements

**Figure 6:**
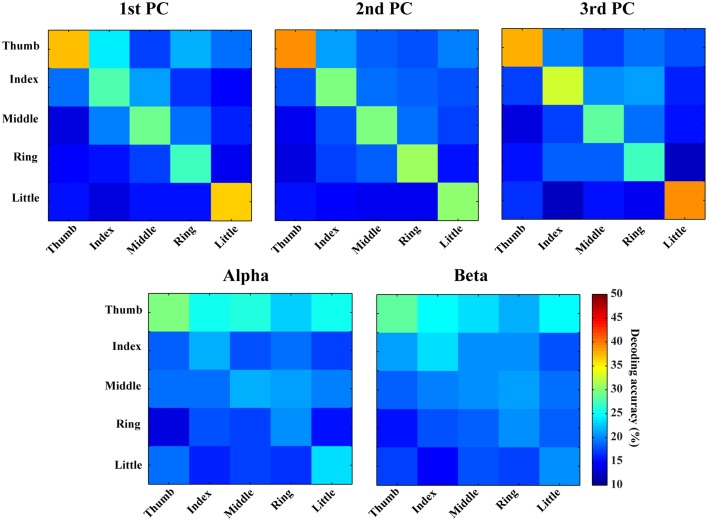
**Confusion matrices of finger movement decoding using individual spectral features**. Each row indicates predicted labels and each column indicates true labels.

**Figure 7:**
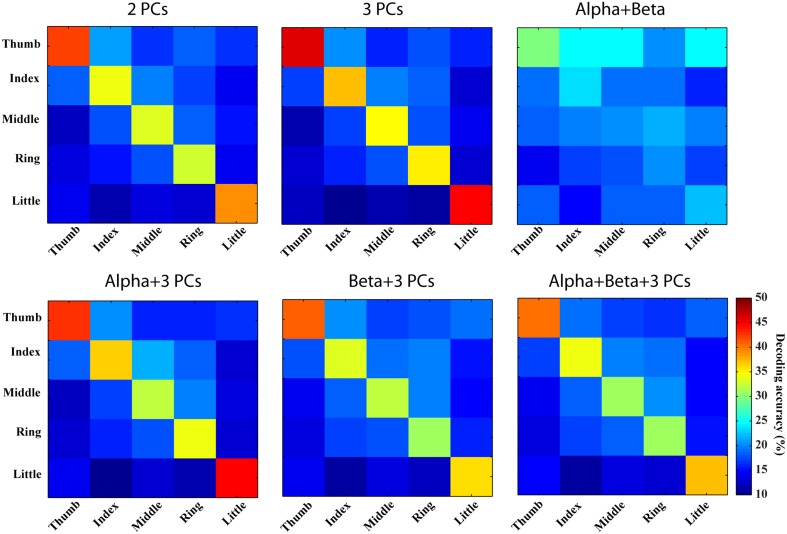
**Confusion matrices of finger movement decoding using combined spectral features**. Row and column labels are same as in Figure 6.

In Figures 6, 7, confusion matrices of five fingers movements from individual or combined spectral features are illustrated. The rows of these matrices stand for predicted condition labels, while the columns represent actual condition labels. For features from individual PCs, similar performances are achieved in all individual PCs and actually moved fingers were dominantly and correctly identified in the confusion matrices (diagonal elements with larger values than off-diagonal elements). Furthermore, the misclassifications are spread almost evenly in four fingers other than the actual one (off-diagonal elements with similar low values). Considering different fingers, thumb and little seem usually better classified than other fingers. For features from alpha and beta bands, only thumb is classified with relatively high accuracies, while the decoding accuracies of other fingers are close to the guessing level (i.e., 20%). Moreover, other four fingers are all confused to thumb, which might be the reason for thumb having high decoding accuracy. Spectral features from PCs show obvious better performance than features from mu/beta PSDs (best mean decoding accuracy in each category: 33.1 vs. 23.4%).

Combinations of spectral features from PCs yield better decoding performance than spectral features from individual PCs (best mean decoding accuracy: 39.7%), while combination of features from mu/beta PSDs does not indicate obvious improvement (best mean decoding accuracy: 23.3%). As shown in Figure 7 (the first row), values of diagonal elements in the confusion matrices from three PCs are further increased, which leads to less confusion among fingers (inferring actual labels are better classified). Similarly, better decoding performances are achieved in thumb and little than other three fingers. On the other hand, the combination of mu/beta PSDs still confuses all five fingers to thumb. The combinations of features from two categories actually show slightly more confusion among fingers than the combination of three PCs (38.1 vs. 39.7%), as shown in Figure 7 (the bottom row).

## Discussion

In the present study, we investigated spectral structures in non-invasive EEG during motor tasks of individual finger movements. Three spectral structures were extracted through a PCA analysis from EEG data, which suggested coupled spectral components over wide (e.g., 1st PC) and/or multiple frequency bands (e.g., 2nd PC). The spatial patterns of these newly identified spectral structures were examined and compared to classic mu/beta rhythms. The resolution of these new spectral features in detecting movements from resting and decoding individual finger movements was further studied in a classification scheme. Our experimental results demonstrate that these new spectral structures from the PCA analysis indicate consistent and specific motor-related spatial patterns in different conditions and subjects. Furthermore, spectral features derived from these new spectral structures are able to reveal discriminative information in non-invasive EEG that is related to fine body-part movements, i.e., finger, beyond large body-part movements (such as hand and shoulder) that can be decoded using classic motor rhythms (i.e., mu and beta rhythms).

### Spectral structures and features

The spectral structures in EEG decomposed by the PCA analysis present different profiles along frequency axis, yet consistency can be found across all channels, conditions, and subjects. These observations are in line with the findings from ECoG studies (Miller et al., 2009), demonstrating the existence of cross-frequency spectral structures in human EEG measured at both the brain and scalp surfaces. Particularly, the first spectral structure (1st PC) suggests a broadband non-rhythmic spectral pattern, which is different from other two spectral structures. The other two (2nd and 3rd PCs) indicate dominant spectral powers at alpha and beta bands, which might resemble rhythmic activities from classic motor rhythms in mu and beta bands (Pfurtscheller and Lopes da Silva, 1999), while other aspects of these two spectral structures, i.e., spatial pattern and resolution in decoding movements, suggest similarity and difference at the same time.

The spatial patterns of these new spectral structures over the channel domain (Figure 2) and their spatial difference patterns between different conditions (e.g., movement vs. resting) (Figure 3) suggest that their activity and activity changes are related to motor brain functions, covering the premotor cortex for movement planning (Hoshi and Tanji, 2000), M1 for movement execution (Stippich et al., 2002), and the posterior parietal cortex (PPC) for integrating sensory and motor information (Fogassi and Luppino, 2005). Their capability in decoding movements (see section below) adds further evidences in linking these new patterns/features to motor brain functions. However, it is unknown, so far, about neural mechanisms behind these spectral structures, especially the broadband non-rhythmic one, while neural mechanisms of rhythmic brain activities have been well investigated (Pfurtscheller and Lopes da Silva, 1999; Urgen et al., 2013). Of course, the rhythmic nature of the 2nd and 3rd PCs (across multiple frequency bands) and their spatial similarity at certain levels to classic mu/beta rhythms might suggest common underlying neural sources among them, while these new spectral structures from the PCA analysis might reveal more coupling and coordinating patterns across different rhythmic activities that cannot be revealed by classic frequency band analysis.

### EEG resolutions in detecting and decoding individual finger movements

EEG resolutions in fine body-part movements have not been sufficiently studied, due to the challenges of limited spatial resolution and SNR in EEG signals (Hassanien and Azar, 2014). Several studies explored the resolution of EEG in decoding finger movements from different hands with accuracies ranging from 70 to 90% (Li et al., 2004; Lehtonen et al., 2008; Wang and Wan, 2009). However, to our knowledge, very few studies have been conducted to decode movements of finger from one hand using EEG. Our present results suggest that features from spectral PC structures can detect finger movements from resting condition with the accuracy up to 86.77% (1st PC), which is significantly better than the accuracy achieved with classic mu/beta rhythms (about 70%). Our confusion matrix analysis further indicates that movements of individual fingers from one hand can be dominantly labeled to correct fingers (outstanding diagonal elements in confusion matrices) using new spectral features from single-trial EEG data, while all fingers are confused to thumb when classic mu/beta rhythmic features are used. It is also important to note that dominantly correct labeling using new spectral features for all fingers is achieved upon the fact that various fingers, especially those close to each other and in the middle, show behavior dependences during movements (Häger-Ross and Schieber, 2000). Thumb is the most independent finger in behaving, which is consistent with our results that thumb is the one with the least confusion (Figures 6, 7). These facts indicate that some confusion is from inherent characteristics of the human motor system. The discriminative information obtained from the PCA analysis on EEG regarding different fingers from one hand suggests that non-invasive EEG can be used to study fine body-part movements beyond large body-part movements that have been well studied using classic rhythmic brain activity (Neuper and Pfurtscheller, 1996; Hashimoto and Ushiba, 2013).

While features from new spectral structures demonstrate that EEG contains information about finger movements, the capability in decoding them in single-trial EEG is still suboptimal. Several factors could be culprits and are worth exploring for improvements. Although the SVM classifier implemented in the present study has been widely adopted, it has been reported that linear program machine (a sparse SVM algorithm) can outperform regular SVM in similar decoding tasks using ECoG signals (Shenoy et al., 2007). A search for more robust decoding algorithms could facilitate the thorough evaluation of new spectral structures and their decoding efficacy in finger movements. EEG signals are known to be susceptible to noises, such as, from ambient environments, motion artifacts, and many others. While spatial CAR filtering and ICA are used to improve SNR in EEG in the present study, other advanced signal processing methods, e.g., common spatial pattern (Ramoser et al., 2000) and stochastic resonance (Lin et al., 2008), can be integrated to further improve EEG signal quality. Another factor might originate from the decoding task of ipsilateral finger movements itself. Movements of individual fingers are usually accompanied with concurrent movements of other fingers, due to muscle connections, tendon organization, and neural control distribution in the hand (Häger-Ross and Schieber, 2000). It is still unclear whether these concurrent movements of uninstructed fingers contribute to misclassifications in the confusion matrices (Figure 6), which requires further efforts to understand the effect from the aspect of behavior correlation.

### Information independence and redundancy in spectral features

Various combinations of spectral features (from new spectral structures and mu/beta rhythms) were studied to probe information independence/redundancy within and cross feature categories. Combinations of PCs can increase the decoding accuracy of movements from resting up to 91%, which is significantly higher than individual PCs (*p* < 0.05). This is also comparable to a recent ECoG study achieving an average of 94% classification rate in detecting any finger movements from resting (Chestek et al., 2013), considering ECoG offers much better signal quality than EEG (Ball et al., 2009). In term of finger decoding, confusion matrices are less confused with more single-trial EEG data correctly labeled (Figure 7). For mu/beta rhythms, combination of alpha and beta PSDs also significantly improves accuracy in detecting movements from resting over individual frequency band PSDs (*p* < 0.05). However, their combination is not able to improve the performance of labeling different fingers, which is reasonable since both lack discriminative information in distinguishing fingers when used alone. These results suggest that different features within each category exhibit independent information in discriminating movements, to which they are sensitive.

Results from cross-category combinations of features, however, suggest no significant improvements in detecting movements from resting (Figure 5), which might suggest that most discriminative information about movements from resting revealed in mu/beta rhythms are also revealed in PCs. Since mu/beta rhythms present little efficacy in decoding fingers, it is expected that cross-category combinations of PCs with mu/beta features would not lead to improvement of finger decoding performance. On the contrary, slight degeneration is observed (Figure 7), which might be attributed to the non-specific nature of mu/beta features to fingers that smoothes out other finger-specific features in the spectral PC structures.

### Implications to BCI applications and neuroprosthesis

Motor rhythm-based BCIs have recently gained increasing attention for its merit of providing asynchronous control on a single-trial basis (Pfurtscheller et al., 2005; Leeb et al., 2007), while most of other popular BCI schemes require repetition of trials for accurate control, such as P300 (Sellers et al., 2006; Mak et al., 2011) and steady state visually evoked potentials (SSVEP, Wang et al., 2008; Bin et al., 2009). However, limited control signals generated from decoding large body parts using classic motor rhythms largely confine the complexity of non-invasive BCI techniques. Until now, such BCIs are only applied to simple applications, such as cursor movements on the computer screen (Wolpaw and McFarland, 2004; Wilson et al., 2009). In the present study, new spectral features present promising movement detection capability and sensitivity to movements of fine body parts, i.e., fingers. With the potential to decode gestures in the future, these new features could provide an alternative mean to overcome the restriction. To be used in neuroprosthesis, they could not only increase the degree-of-freedom of control signals, but also contribute to a more naïve mapping from EEG to robotic fingers. Further, robust detection of movements from resting can create an idle control state, which is crucial in designing online applications for both BCI and neuroprosthesis (Blankertz et al., 2002). It is, however, important to note the decoding performance has yet to reach the level of practical usage. Our present study only demonstrated the feasibility in decoding movements of fine body parts using non-invasive EEG recordings. Its practical usage in the future is expected to be dependent on significantly refined detection of usable signals and significantly improved classification accuracy.

### Conflict of interest statement

The authors declare that the research was conducted in the absence of any commercial or financial relationships that could be construed as a potential conflict of interest.
